# High-resolution optical design of the sub-meV ARPES beamline at the Ultrafast Transient Experimental Facility

**DOI:** 10.1107/S1600577525007556

**Published:** 2025-09-17

**Authors:** Tao Lei, Chaoyang Li, Baiqing Lv, Mojun Pan, Bocheng Jiang, Hong Ding, Dao Xiang

**Affiliations:** a Laboratory for Ultrafast Transient Facility Chongqing University Chongqing People’s Republic of China; b Anhui Specreation Instrument Technology Co. Ltd, Anhui, People’s Republic of China; c School of Physics and Astronomy and Tsung-Dao Lee Institute Shanghai Jiao Tong University Shanghai People’s Republic of China; Australian Synchrotron, Australia

**Keywords:** ARPES beamline, varied-line-spacing plane-grating monochromator, synchrotron radiation, EUV beamline design

## Abstract

The Ultrafast Transient Experimental Facility (UTEF) at Chongqing University is constructing a next-generation angle-resolved photoemission spectroscopy (ARPES) beamline designed to simultaneously achieve sub-meV energy resolution, continuous photon energy tunability (10–40 eV), high photon flux (>10^12^ photons s^−1^ at the sample position), full polarization control, and ultra-low-temperature sample environments (<1.5 K). Leveraging the unique advantages of UTEF’s low-energy (0.5 GeV), high-beam-current (500–1000 mA) storage ring, the beamline is capable of generating high-flux EUV radiation, enabling detailed exploration of complex quantum materials. The beamline employs two high-groove-density gratings and one low-groove-density grating to achieve, respectively, an energy resolving power exceeding 100000 and photon fluxes greater than 5×10^13^ photons s^−1^. A dual-endstation layout enables flexible operation for both ultra-high-resolution measurements at ultra-low temperatures and large-angle, high-flux spin-resolved experiments. Through comprehensive optical optimization—including mirror coatings, customized grating groove profiles and precision focusing geometry—the system can deliver photon flux exceeding 10^14^ photons s^−1^ (0.1% bandwidth)^−1^ with a spatial beam spot size of approximately 30 µm, while maintaining sub-0.4 meV energy resolution. This work presents the optical design and projected performance of the UTEF ARPES beamline.

## Abstract 2

The Ultrafast Transient Experimental Facility at Chongqing University is developing an advanced ARPES beamline capable of 0.4 meV energy resolution within the 10–40 eV photon energy range, featuring photon flux over 10^12^ photons s^−1^, tunable polarization and ultra-low temperatures below 1.5 K. Leveraging a 0.5 GeV, 500–1000 mA storage ring, the design employs a dual-endstation setup for ultra-high-resolution and high-flux spin-resolved experiments.

## 1. Introduction

Angle-resolved photoemission spectroscopy (ARPES) is a powerful technique capable of directly probing the energy, momentum and spin information of electrons in solids, playing an irreplaceable role in the study of high-temperature superconductors, topological quantum materials, and other strongly correlated systems (Lv *et al.*, 2015 ▸; Li *et al.*, 2020 ▸; Lei *et al.*, 2022 ▸). Among ARPES methods, laser-based ARPES, with photon energies typically in the 7–9 eV range, has demonstrated remarkable energy resolution and made significant contributions in the study of superconducting gap structures (Liu *et al.*, 2008 ▸; He *et al.*, 2016 ▸). However, due to the intrinsically limited photon energy of lasers, such systems suffer from restricted momentum-space coverage and are thus inadequate for comprehensive investigations of many quantum materials.

In contrast, synchrotron-radiation-based ARPES offers tunable photon energies ranging from a few electron volts (eV) to several thousand eV, enabling access to a much broader momentum space and deeper energy level (Strocov *et al.*, 2014 ▸). Nevertheless, existing synchrotron-based ARPES facilities typically achieve energy resolutions only at the level of several millielectronvolts (meV), which falls short of the ultra-high precision demanded by studies on fine energy scales and subtle electronic states (Hoesch *et al.*, 2017 ▸; Varykhalov, 2018 ▸). For instance, probing electronic structures near the (π, π) point or the *M* point in materials like magic-angle twisted bilayer graphene often requires photon energies exceeding 20 eV—beyond the reach of current laser-ARPES systems (Chen *et al.*, 2024*a* ▸). Moreover, many quantum materials possess three-dimensional electronic structures, necessitating broad-range, continuously tunable photon energy to fully resolve the three-dimensional Brillouin zone (Chen *et al.*, 2024*b* ▸).

The development of next-generation ARPES beamlines with both high photon energy and ultra-high energy resolution is therefore crucial (Borisenko, 2012 ▸). To address these demands, the ARPES beamline at the Ultrafast Transient Experimental Facility (UTEF) of Chongqing University, China, has been meticulously designed to achieve a comprehensive energy resolution of 0.4 meV. This is realized by leveraging the advantages of UTEF’s electron beam, which features a relatively low energy of 0.5 GeV and a high current of 500–1000 mA, making it ideal for generating high-flux extreme ultraviolet (EUV) radiation. Through the optimization of the beamline optics and the implementation of an ultra-low-temperature sample environment, the designed ARPES system aims to significantly surpass the performance of existing facilities in the 10–40 eV photon energy range (Borisenko, 2012 ▸; Lagarde *et al.*, 2013 ▸; Kimura *et al.*, 2010 ▸).

Furthermore, considering the trade-off between monochromator resolving power and photon flux, the UTEF ARPES beamline ensures a photon flux of at least 10^12^ photons s^−1^ at the sample position, even when operating with a monochromator resolving power exceeding 100000. This corresponds to an upstream photon flux of 1.0 × 10^16^ photons s^−1^ (0.1% bandwidth)^−1^ at 25 eV, surpassing the capabilities of current leading synchrotron sources by an order of magnitude. Additionally, to fully utilize the ultra-high energy resolution, the sample temperature must be maintained below 1.5 K, as thermal broadening would otherwise obscure the fine electronic features on the meV scale. In this work, we present the optical design and expected performance of the high-resolution ARPES beamline under construction at UTEF, aiming to establish a world-class facility for precision electronic structure studies of quantum materials.

## 2. Storage ring and undulator source

The UTEF of Chongqing University is the city’s first large-scale scientific facility. It is currently constructing a low-energy, high-current synchrotron source comprising a 0.5 GeV linear accelerator and a high-current storage ring. The storage ring employs innovative technologies to achieve low emittance and high beam current, including the use of low magnetic field strengths combined with enlarged vacuum chamber apertures to reduce impedance, as well as the application of a third harmonic cavity to lengthen the bunch length and mitigate collective effects (Bosch *et al.*, 2001 ▸). The storage ring has a circumference of 76.78 m and operates at a typical beam current of 1000 mA. It features three straight sections designated for the installation of insertion devices. The main parameters of the storage ring are summarized in Table 1 ▸.

**Figure 1:**
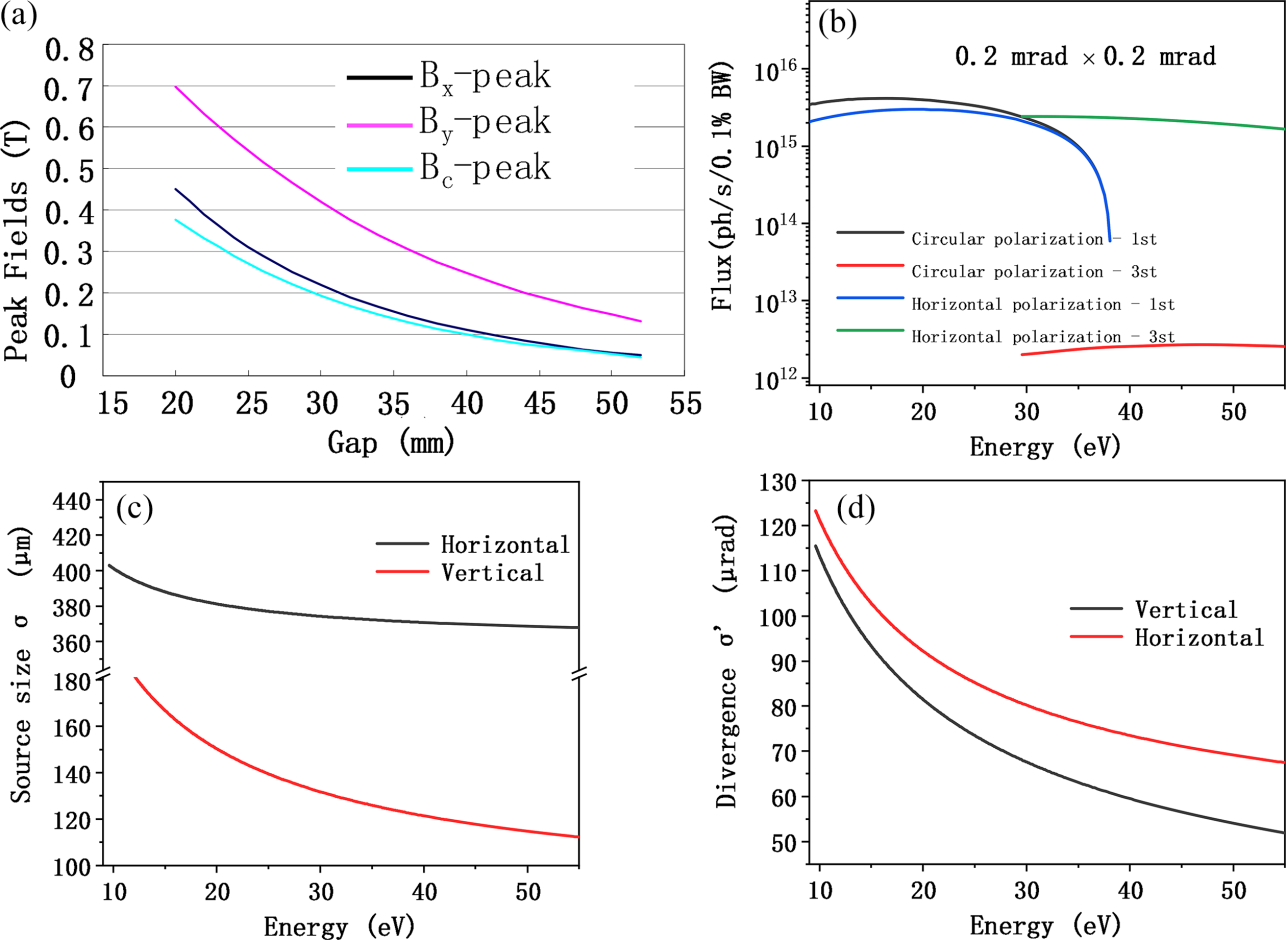
(*a*) Variation of the peak magnetic field as a function of the gap for the three palarization modes; (*b*) estimated photon flux within a collection solid angle of 0.2 mrad × 0.2 mrad of the undulator in linear and circular polarization modes; (*c*) effective source size and (*d*) angular divergence of the undulator radiation.

The ARPES beamline is fed by radiation from an APPLE-II type helical polarization undulator (EPU), which has been parameter-optimized to provide high-flux, tunable-polarization photons in the energy range 10–40 eV (Sasaki, 1994 ▸). The undulator has a period length of 62 mm and consists of 78 periods, resulting in a magnetic structure length of 4.867 m and a total device length of 5.0 m. The minimum magnetic gap is 20 mm, enabling a minimum photon energy of 7 eV in circular polarization mode. Fig. 1 ▸(*a*) shows the peak magnetic field as a function of the gap for three different polarization modes. The EPU contains a total of 311 standard-sized permanent magnet blocks per array, with 156 horizontally magnetized and 155 vertically magnetized. Additionally, each end of the array is equipped with three permanent magnets forming the end magnets, of which two are vertically magnetized and the central one is horizontally magnetized. All four magnetic arrays are capable of longitudinal translation, allowing for full control over the polarization state of the emitted light. The longitudinal shift adjustment precision is better than 25 µm, while the gap adjustment precision is within 5 µm. The main parameters of the undulator are summarized in Table 2 ▸. Compared with medium- and high-energy storage rings, the 0.5 GeV low-energy storage ring at UTEF is capable of generating EUV radiation using a short-period undulator. For a given straight section length, the undulator accommodates a larger number of periods, which, when combined with the high beam current characteristics of the storage ring, results in significantly enhanced photon flux output. Moreover, the use of low-energy electrons substantially suppresses the total radiative power. According to calculations performed using the *SPECTRA* code, the maximum total power emitted by the insertion device is estimated to be approximately 150 W (Tanaka & Kitamura, 2001 ▸). After attenuation by the white-light shutter and a four-blade slit system, the power incident on the first optical element is reduced to around 3 W, and the absorbed power at the monochromator’s plane mirror is less than 2 W. The ability to generate intense EUV radiation while maintaining low thermal load on optical components is critical for achieving sub-meV energy resolution, as it minimizes thermally induced mirror deformation and optical instability.

The electron beam size and divergence are convoluted with the diffraction-limited size and divergence to derive the effective source parameters in undulator mode. The actual root-mean-square (r.m.s.) source size and divergence are calculated using the following formulas: σ_*r*_ = 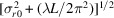, 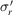 = 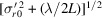, where σ_*r*0_ and 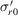 represent the intrinsic electron beam size and divergence, respectively; *L* denotes the undulator length, and λ is the emitted wavelength (Sasaki, 1994 ▸). The effective source size and divergence obtained from these calculations are shown in Figs. 1 ▸(*c*) and 1(*d*).

To enhance beamline efficiency and accommodate different experimental requirements, a dual-endstation design has been adopted. One experimental station (endstation B) is dedicated to ultra-low temperature, high-energy-resolution measurements, while the other (endstation A) focuses on high-flux, spin-resolved measurements. The high-resolution mode covers a photon energy range of 10–40 eV, whereas the high-flux mode operates within the 20–40 eV photon energy range.

## 3. Optical design

To achieve an energy resolving power exceeding 100000 in the 10–40 eV range, the use of an off-plane Eagle normal incidence monochromator (NIM) is considered as one possible option (Koike *et al.*, 1994 ▸; Heimann *et al.*, 1997 ▸; Petaccia *et al.*, 2009 ▸). Internationally, there have been successful implementations of this design, where replication gratings have achieved resolutions of up to 70000, and precision-manufactured gratings have reached resolving powers exceeding 100000 for photon energies below 20 eV. However, these high resolutions have been obtained using gratings with very high groove densities, typically around 4300 lines per millimetre (l/mm) or 4800 l/mm (Heimann *et al.*, 1997 ▸; Nahon *et al.*, 2001 ▸). Although the NIM configuration is favored for its capability to provide high resolution over a broad photon energy range—unlike grazing incidence monochromators, which achieve high flux and high resolution only within a narrow energy range due to the strong dispersion of coma aberrations—there are notable drawbacks (Eyers *et al.*, 1983 ▸). One of the main disadvantages of the NIM design is that, when tuning photon energy, the exit beam direction changes horizontally after the exit slit (Schäfers *et al.*, 1986 ▸; Cash, 1982 ▸). This necessitates adjustments to downstream optical components to maintain a constant beam spot position on the sample. For large storage rings of third-generation synchrotron sources, where orbit stability and reproducibility are ensured by advanced feedback systems, such adjustments are manageable. The downstream mirrors can be pre-calibrated, and their positions can be set according to the photon energy with high precision. However, for smaller storage rings like UTEF, orbit stability and reproducibility are generally less robust. This could lead to significant alignment challenges for the optical system beyond the exit slit during practical operation.

Moreover, the diffraction efficiency of NIM-type gratings is relatively low, which reduces the overall beamline flux compared with grazing incidence monochromator configurations (Cash, 1982 ▸; Poletto & Frassetto, 2009 ▸). Taking these factors into account, the optical design of this beamline adopts a varied-line-spacing plane-grating monochromator (VLS-PGM) scheme, similar to the Dreamline at the Shanghai Synchrotron Radiation Facility (SSRF) (Xue *et al.*, 2014 ▸).

Given that the vertical source size of the UTEF light source is relatively large, approximately 0.2 mm, if a 3600 l/mm grating is used with a included angle of 145°, the monochromator would need to be positioned more than 30 m away from the source to achieve a resolving power of 100000 based solely on source size constraints. However, this distance exceeds the spatial limitations of the experimental hall. Therefore, a prefocusing mirror will be employed to demagnify the source onto the entrance slit, effectively creating a secondary source point for the monochromator (Lei *et al.*, 2023 ▸). Utilizing a secondary source offers the advantage of mitigating the effects of beam jitter and discrepancies between the actual and theoretical source sizes on the energy resolution.

**Figure 2:**
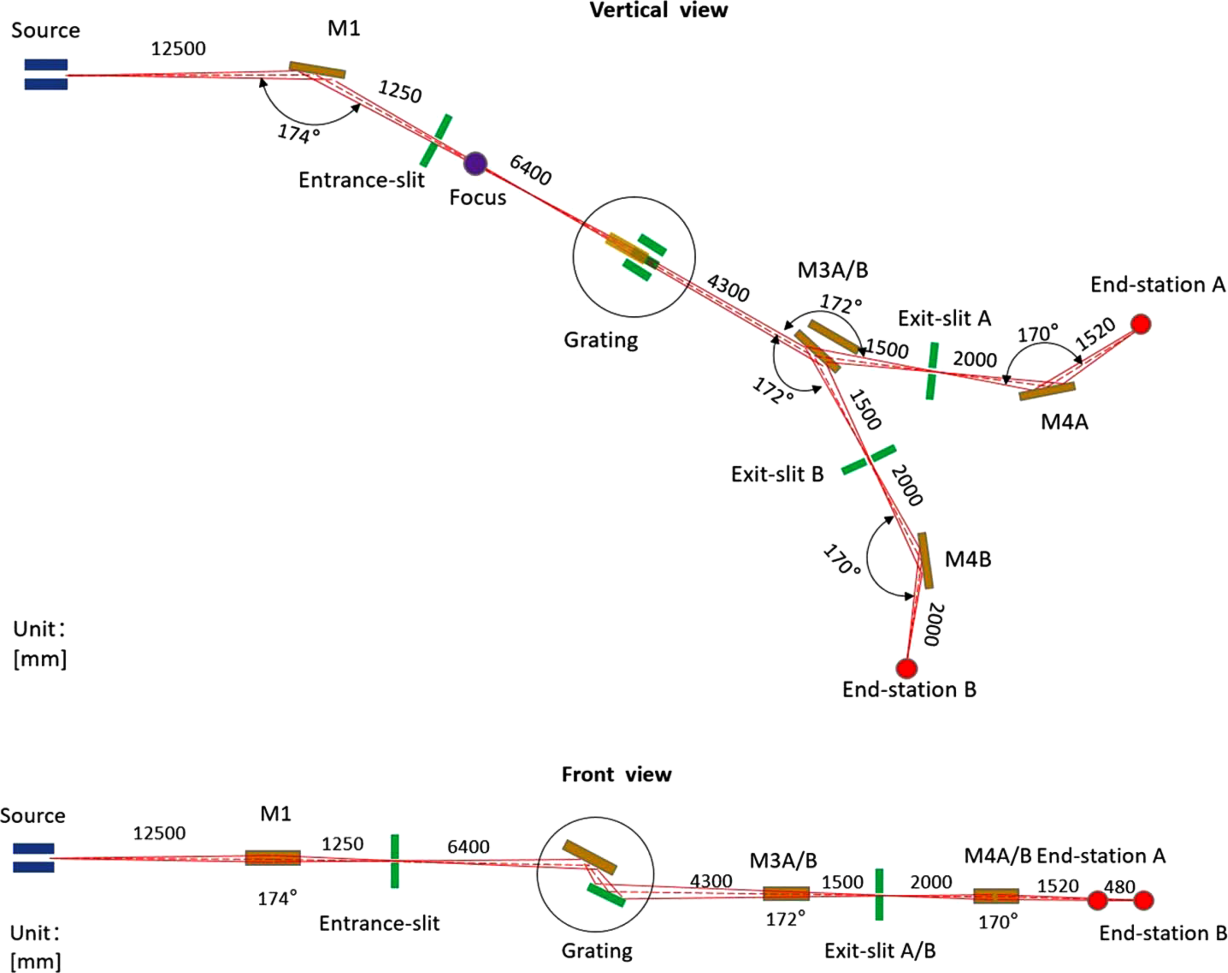
Optical layout of the high-energy resolution ARPES beamline at UTEF.

The overall optical layout of the beamline is shown in Fig. 2 ▸. Mirror M1 is a toroidal mirror positioned 12.5 m downstream from the source point, operating at a grazing incidence angle of 3°. It focuses the beam vertically onto the entrance slit with a demagnification ratio of 10:1. This design serves two purposes: first, it allows for adjustment of the slit size to control the contribution of the source term to the overall energy resolution; second, it increases the vertical divergence angle of the beam by approximately tenfold, thereby providing sufficient illumination for the grating and reducing the impact of diffraction-limited effects on energy resolution. Additionally, this helps to keep coma aberrations within an acceptable range. In the horizontal plane, M1 focuses the beam to a point located 0.5 m downstream of the entrance slit, effectively minimizing aberrations and reducing the required dimensions of the downstream focusing optics. To meet the diverse experimental needs of different material systems and research objectives, the beamline is configured with two endstations: endstation B is dedicated to ultra-low temperature, high energy resolution measurements, while endstation A is designed for high-flux, large-angle spin-resolved measurements. Correspondingly, the beamline employs a grating set optimized for both high-flux and high-resolution modes. A low groove density grating with 500 l/mm (VPG3) ensures the photon flux required for spin-resolved measurements, while higher groove density gratings of 1400 l/mm (VPG2) and 2800 l/mm (VPG1) are used to provide narrow-bandwidth radiation necessary for high energy resolution measurements (Padmore, 1989 ▸). The monochromator adopts a VLS-PGM configuration, consisting of a vertically downward-reflecting plane mirror (M2) and three separate upward-facing grating. This arrangement brings the beam height down from 1300 mm orbit plane to the 1260 mm sample height. The three VLS gratings are specifically designed and optimized with groove densities of 2800 (VPG1), 1400 (VPG2) and 500 l/mm (VPG3), enabling full coverage of the required photon energy range and facilitating mode switching between high-resolution and high-flux operations. VPG3 is optimized for the 17–40 eV range, while VPG1 and VPG2 are optimized for the 19–40 eV and 9.5–20 eV ranges, respectively. Detailed specifications of these gratings are provided in Table 3 ▸.

**Figure 3:**
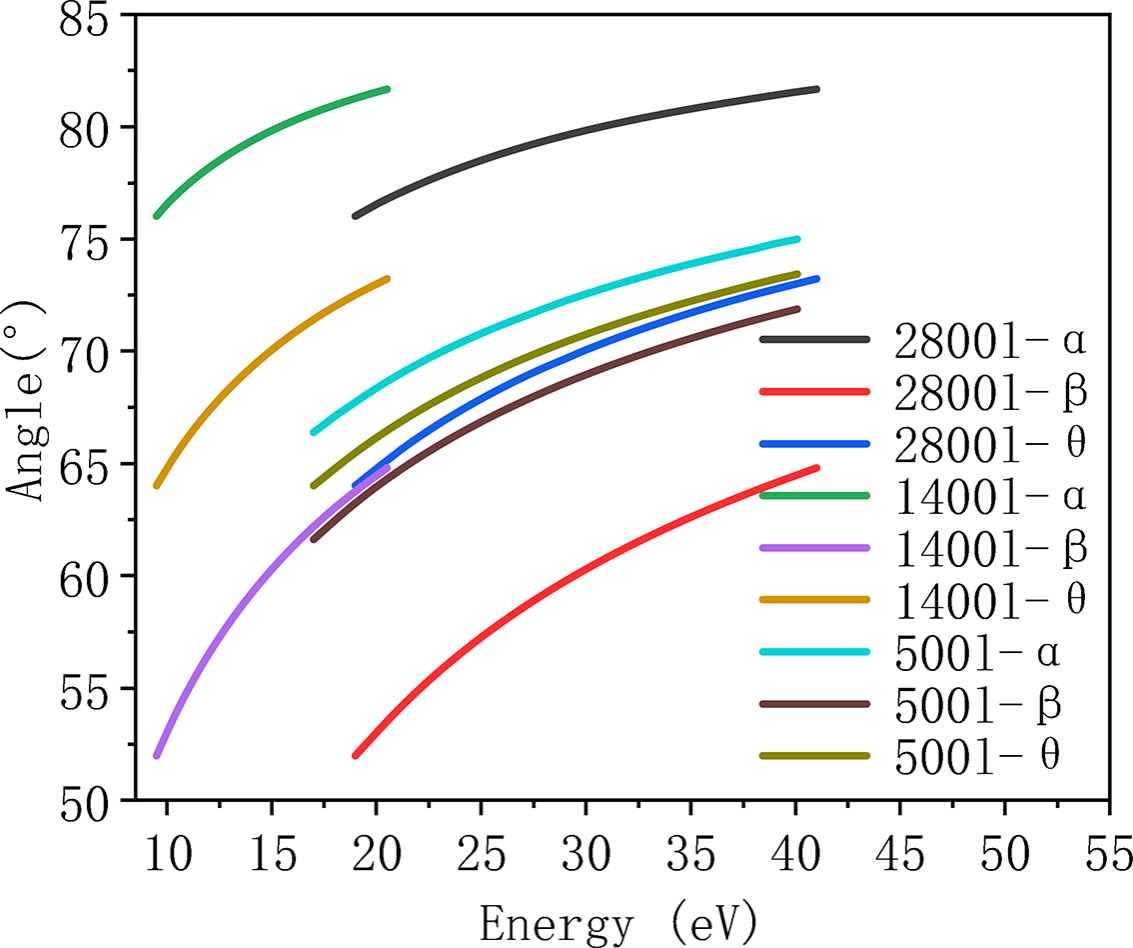
The angular dependence of the grating and plane mirror as a function of photon energy.

Mirror M2 requires off-axis rotation to adjust the included angle, ensuring that the incident light consistently illuminates the center of the selected grating while maintaining focus at the exit slit. The included angles of the gratings range from 128° to 146.5°. For VPG1 and VPG2, the required grating rotation is 12.82°, with a corresponding M2 rotation of 9.23°. For VPG3, the grating rotation is 10.27°, and M2 rotates by 9.43°. All these values are within the allowable limit of the mechanical design. Fig. 3 ▸ illustrates the relationship between the incident angles of the plane mirror and the three gratings as a function of photon energy. The VLS-PGM design integrates both monochromatization and focusing functionalities, which are not achievable with conventional plane gratings (Reininger, 2011 ▸). By optimizing the polynomial coefficients of the groove density distribution, this design effectively reduces or eliminates specific aberrations in the diffraction direction, thereby enhancing imaging quality and improving energy resolution. It is worth noting that the contributions of source size and exit slit width to the overall energy resolution can be mitigated by positioning the monochromator and exit slit as far downstream as possible. However, this must be balanced against the practical constraints of beamline layout and the limited space within the experimental hall. Taking these factors into consideration, the incident arm and exit arm lengths of the monochromator have been set to 6400 mm and 5800 mm, respectively, ensuring an optimal compromise between resolution performace and spatial feasibility.

At a distance of 4300 mm downstream from the monochromator, two switchable horizontally positioned cylindrical-ellipsoidal mirrors (M3A and M3B) are employed to direct the beam towards two separate endstations. These mirrors focus the beam horizontally onto the exit slit, operating at a grazing incidence angle of 4°. Subsequently, at 2000 mm downstream from the exit slit, each branchline is equipped with an ellipsoidal mirror (M4A for endstation A and M4B for endstation B), which simultaneously focuses the beam in both horizontal and vertical directions onto the respective sample positions. The focal distances for M4A and M4B are designed as 1520 mm and 2000 mm, respectively, with both mirrors operating at a grazing incidence angle of 5°. The combination of M3A/B and M4A/B effectively separates the beam paths to provide sufficient spatial clearance, ensuring ample installation space for the two endstations. The detailed parameters of these optical components are summarized in Table 3 ▸.

## 4. Expected performance

### 4.1. Energy resolution

The VLS-PGM utilizes the principle of diffraction to disperse the incident broadband synchrotron radiation according to wavelength and angle. Compared with conventional constant-line-spacing gratings, the VLS-PGM possesses an inherent self-focusing capability, effectively reducing the number of required optical elements. Additionally, by adjusting the line-spacing parameters, it enables intrinsic compensation of optical aberrations. The groove density distribution of the VLS grating can be expressed by the following polynomial equation,
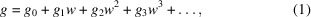
where *g* denotes the local groove density as a function of position *w* along the grating length. Here, *g*_0_ represents the groove density at the grating center (*w* = 0), and *w* is defined as positive in the direction towards the exit slit (Itou *et al.*, 1989 ▸). The grating diffraction condition follows
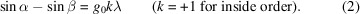
The linear coefficient term *g*_1_ can be optimized to eliminate the defocus term *f*_20_ in the optical path function; when *f*_20_ = 0, the focusing condition is satisfied, expressed as
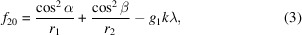
where *r*_1_ and *r*_2_ are the distances from the grating to the object (entrance slit) and image (exit slit) points, respectively, while α and β are the incidence and diffraction angles.

Similarly, the quadratic coefficient term *g*_2_ can be adjusted to eliminate the coma term *f*_30_ in the optical path function at a specific wavelength, given by
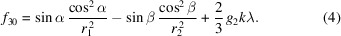
The primary feature of this monochromator design is that the defocus term *f*_20_ is zeroed for all wavelengths by using M2 to illuminate the grating at the required angle of incidence. The coma term is not identically zero for all photon energies but its impact on energy resolution can be minimized through appropriate parameter optimization (Strocov *et al.*, 2011 ▸).

**Figure 4:**
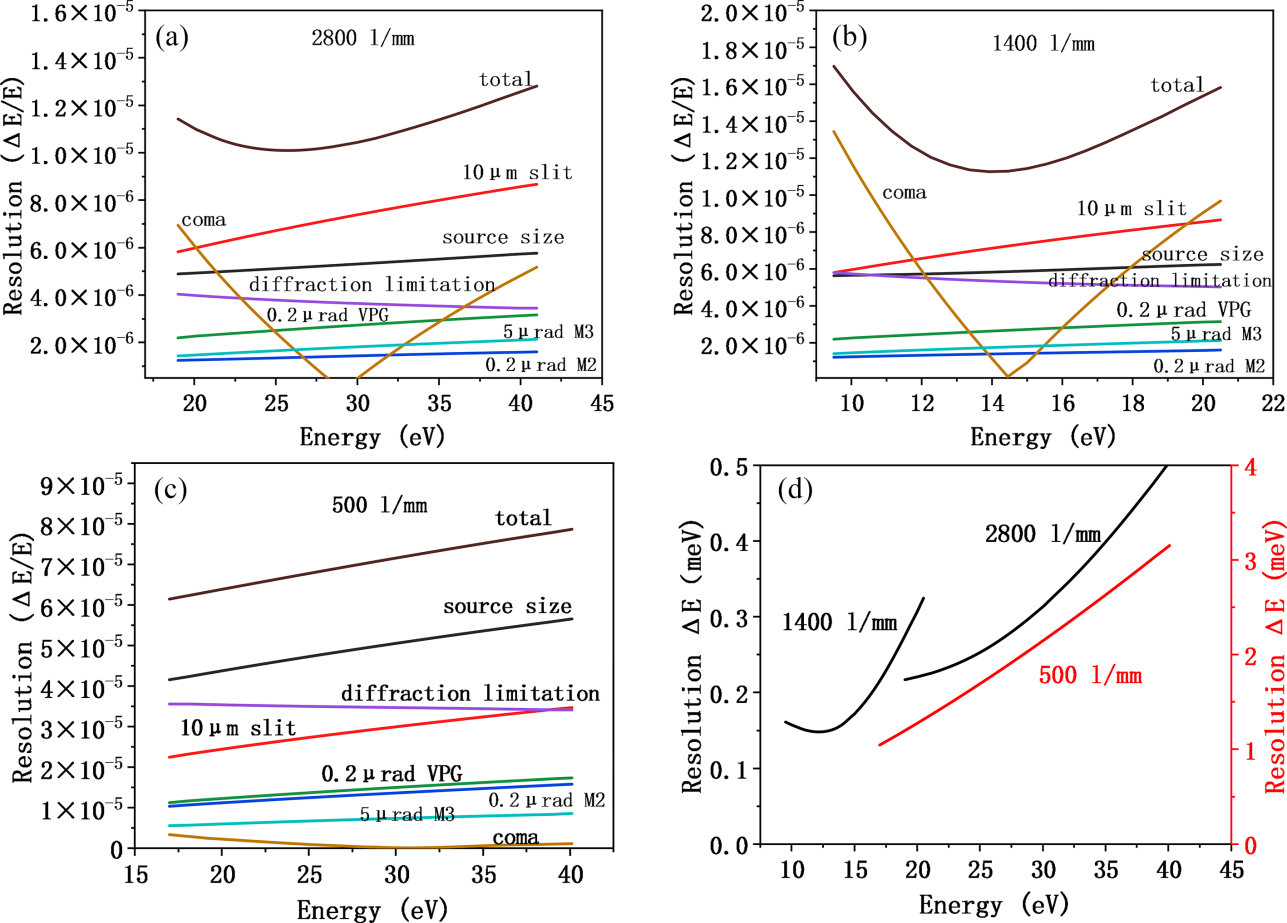
Contributions of individual factors to the energy resolution for VPG1 (*a*), VPG2 (*b*) and VPG3 (*c*); (*d*) overall energy resolution for the three gratings.

The resolving power is defined by the full width at half-maximum (FWHM) expression *R* = *E*/Δ*E*_FWHM_, where two spectral lines are considered resolvable if their separation at the exit slit, in the dispersive direction, equals Δ*E*_FWHM_. The principal contributions to the resolving power originate from several factors, including: the vertical source size (or full entrance slit height, *R*_so_), the full height of the exit slit (*R*_slit_), the sagittal root-mean-square (r.m.s.) slope error of mirror M3 (*R*_M3_), the tangential r.m.s. slope error of mirror M2 (*R*_M2_), the intrinsic contribution of the grating (*R*_Gra_), the coma aberration term (*R*_com_) and the diffraction limit (*R*_diff_). These individual components can be evaluated using the following equations,
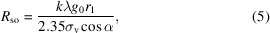

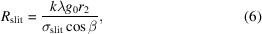

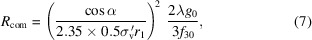

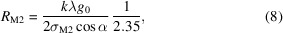

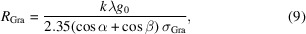

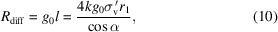

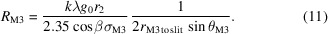
Here, σ_v_, σ_slit_, *l*, 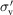, σ_M3_, σ_M2_ and σ_Gra_ represent the vertical photon source size, vertical exit slit width, illuminated grating length, vertical source divergence, sagittal slope error of M3, tangential slope error of M2, and slope error of the grating, respectively. In addition, *g*_0_ is the central line density of the VLS grating, *k* is the diffraction order (taken as 1), θ_M3_ is the incidence angle on M3, and λ is the photon wavelength. The total energy resolution of the beamline is then given by the quadratic sum of these seven contributions,
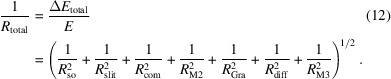
Figs. 4 ▸(*a*), 4(*b*) and 4(*c*) illustrate the contributions of various optical factors to the overall energy resolution for the three VLS gratings. As shown in the figures, for both VPG1 and VPG2, the calculation results indicate that M3, which serves as a horizontally deflecting optical element with a planar surface in the sagittal direction, contributes negligibly to the energy resolution. A slope error of 0.2 µrad for both M3 and the VPG grating is sufficient to meet the required resolution performance. The dominant factors affecting resolution are the vertical source size, the exit slit width, the diffraction limit and the coma aberration, which are intrinsically coupled—primarily influenced by the source size and divergence. Therefore, it is essential that both the entrance and exit slits be easily adjustable and function cooperatively to optimize the trade-off between energy resolution and photon flux. For VPG3, as shown in Fig. 4 ▸(*c*), the influence of the coma aberration becomes negligible. The key resolution-limiting factors are the source size, exit slit width and diffraction limit. Theoretical calculations suggest that, to achieve a desirable balance between resolution (approximately 4 meV) and photon flux, the exit slit width should be set to around 20 µm.

**Figure 5:**
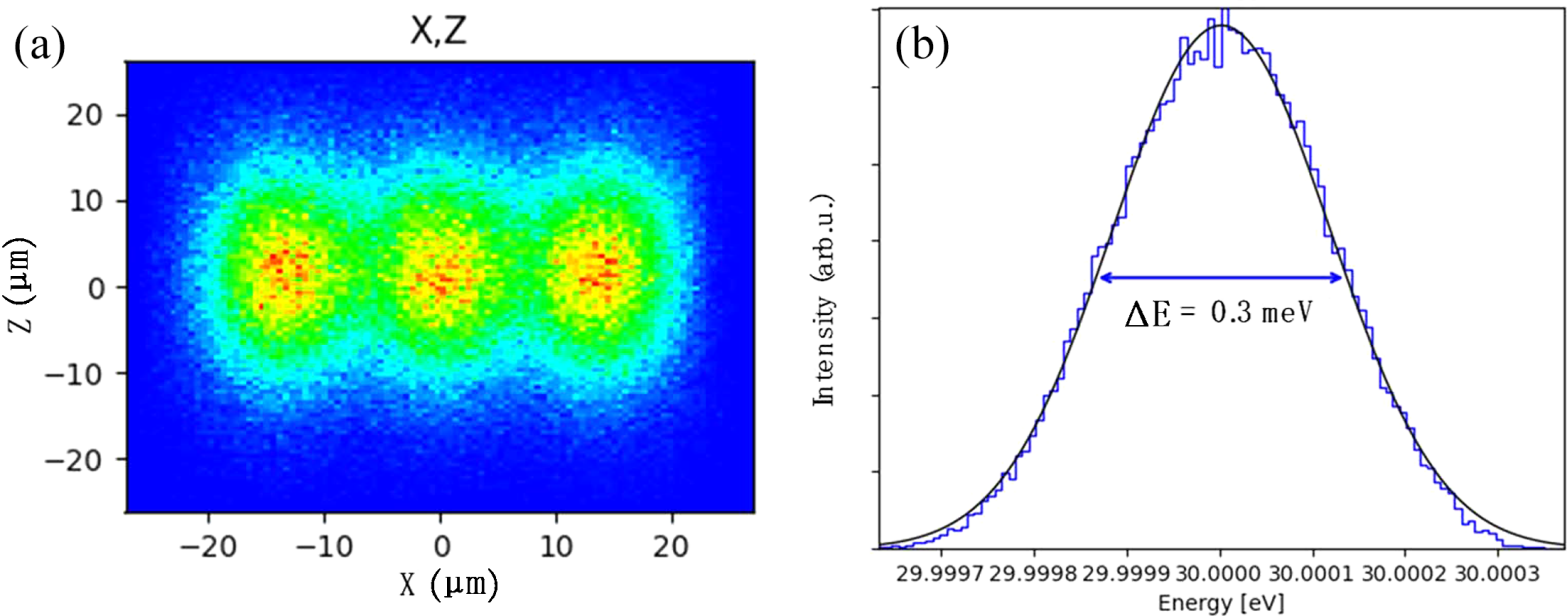
(*a*) Spot patterns on the exit slit plane at 29.9997, 30.0000 and 30.0003 eV with VPG1 confirming the achievable design targets. (*b*) Results of ray-tracing simulation and Gaussian fitting at 30 eV showing that the resolution of VPG1 is 0.3 meV.

Fig. 4 ▸(*d*) presents the simulated resolution over the full energy range under the condition of a fully open entrance slit. The results show that the high-resolution mode can achieve a resolving power corresponding to <0.4 meV across the entire energy range. Furthermore, the design enables resolution tuning by adjusting the slit sizes. If higher resolution is required, it can be further improved by reducing the vertical heights of the entrance and exit slits. These results have been cross-validated using ray-tracing simulations performed with the *SHADOW* code, adopting the same r.m.s. slope error values used in the analytical models (Rebuffi & Sanchez del Rio, 2016 ▸). The simulation output in Fig. 5 ▸(*b*) demonstrates that VPG1 achieves an energy resolution better than 0.3 meV (FWHM) at a photon energy of 30 eV. The Gaussian-like spot profile confirms that optical aberrations are effectively suppressed and have negligible impact on resolution performance.

### 4.2. Reflectivity and mirror efficiency

**Figure 6:**
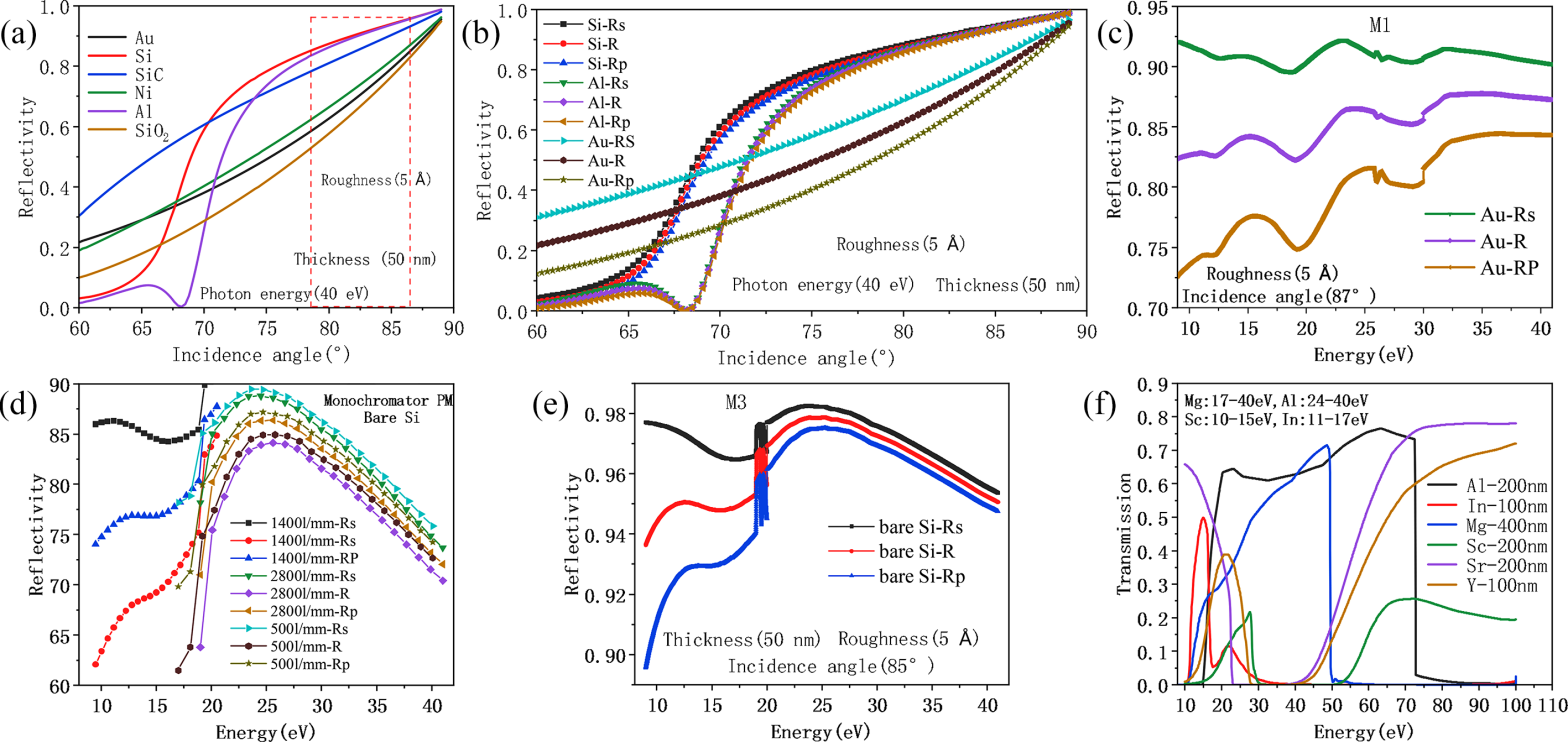
(*a*) Reflectivity of several common coating materials as a function of incidence angle at a photon energy of 40 eV. (*b*) Variation in reflectivity with incidence angle for Si, Au and Al coatings under different polarization states. (*c*) Reflectivity of the M1 mirror coated with Au. (*d*) Reflectivity of the plane mirror M2 in the monochromator. (*e*) Reflectivity of the M3 mirror with a bare Si substrate. (*f*) Transmittance curves of several typical filters as a function of photon energy, with the selected filter materials and their corresponding energy ranges indicated for this beamline.

The efficiency of mirrors is influenced by various factors, including the incident angle of optical components, the characteristics of coating materials, surface roughness, the polarization state of incident light, and the energy of incident light. Fig. 6 ▸(*a*) shows the variation trend of reflectance of several commonly used coating materials such as Au, Al, Si and SiC at different incident angles (Palik, 1998 ▸). It can be seen from the figure that, within the incident angle range selected for this beamline, SiC or bare Si substrates exhibit higher reflection efficiency. Additionally, Si substrates are insensitive to the polarization state of light, while Au coatings show strong polarization dependence [Fig. 6 ▸(*b*)]. Therefore, if the light source needs to adjust the polarization state, choosing Si substrates can ensure that the system maintains a high transmission efficiency. However, the surface of bare Si substrates is prone to oxidation reactions, which can affect their performance. We also calculated the impact of varying Si oxide layer thicknesses on reflectivity at different photon energy; the results indicate that, within the energy range of 10–40 eV, the reflectivity decreases significantly with increasing oxide layer thickness, except in the low-energy segment. Specifically, when the oxide layer thickness is less than 10 nm, reflectivity drops from over 90% to approximately 70–80%. Literature reports indicate that, in ultra-high vacuum environments, the oxidation thickness of Si substrates typically remains in the nanometre range (Horie *et al.*, 1994 ▸), providing a critical reference for evaluating the performance of Si-based mirrors in practical applications.

From the above analysis, it can be seen that, within the energy range of 10–40 eV, Si material is significantly superior to other materials in terms of reflectivity and adaptability to different polarization states. Therefore, in the construction of this beamline, bare Si is preferred for the mirrors as much as possible. To mitigate the formation of oxide layers on Si surface, several engineering measures have been implemented during beamline construction. These include improving vacuum levels within the chamber, conducting pre-conditioning treatments, and minimizing the exposure time of optical elements to ambient air. Taking into account factors such as optical performance, fabrication complexity and overall cost, the first mirror M1 is designed as a toroidal mirror. Since M1 is not readily replaceable or switchable, it is coated with Au film to ensure long-term stability and reflectivity. The reflectance of the Au coating on M1 is shown in Fig. 6 ▸(*c*). For the diffraction gratings, which can be switched in operation, a dual-stripe coating scheme has been adopted. One stripe uses a bare Si substrate to maximize photon throughput, while the other is coated with Au to provide greater long-term stability and resistance to surface degradation. Mirrors M2, M3 and M4 are all fabricated using bare Si to maintain high overall beamline transmission efficiency. Should reflectivity degradation occur due to oxide growth, it can be addressed through *in situ* cleaning procedures or replacement with new mirrors. The reflectivity of M3, as an example, is illustrated in Fig. 6 ▸(*e*).

### 4.3. Transmission efficiency and photon flux

**Figure 7:**
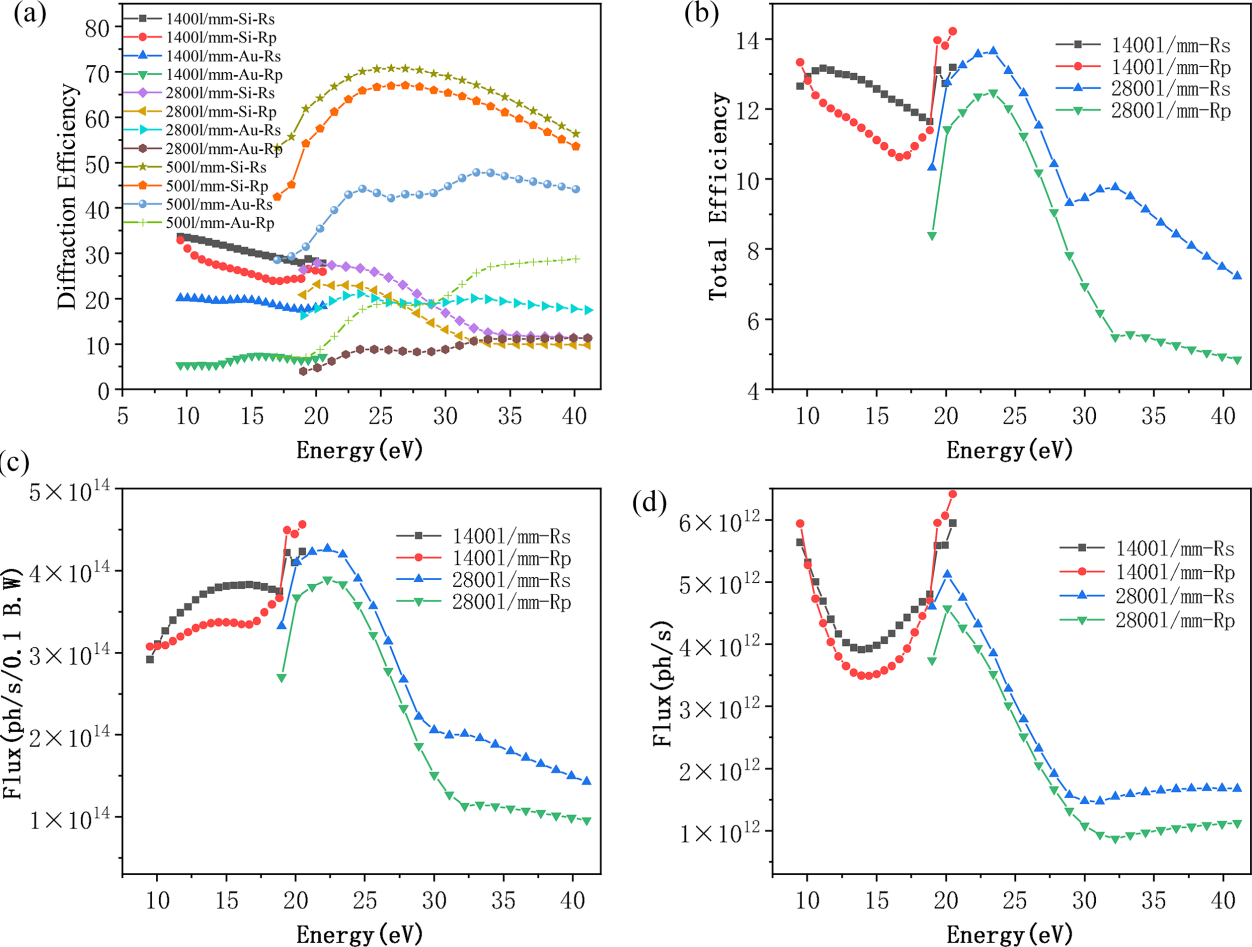
(*a*) Diffraction efficiency of the three gratings. (*b*) Overall transmission efficiency of the beamline. (*c*) Beamline photon flux within a 0.1% bandwidth. (*d*) Total photon flux of the complete system.

The total transmission efficiency of the beamline can be expressed as the product of three components: the geometric transmission efficiency, the mirror reflectivity and the grating diffraction efficiency (Lei *et al.*, 2023 ▸). Among these, the geometric transmission efficiency is primarily determined by the acceptance angle of the optical system and the photon transmittance through the entrance and exit slits. Fig. 7 ▸(*a*) presents the calculated diffraction efficiency of the gratings. For the 2800 l/mm grating, the Si-coated stripe exhibits superior diffraction efficiency compared with the Au-coated stripe when the photon energy is below 30 eV, whereas the Au-coating stripe becomes more efficient at photon energies above 30 eV. By selecting the appropriate coating stripe, the theoretical diffraction efficiency of VPG1 is estimated to range from 10% to 30%. For the 1400 l/mm grating, the Si-coated stripe consistently outperforms the Au-coated strip, with efficiency values ranging from 23% to 35%. Similarly, for the 500 l/mm grating, the Si-coated stripe provides higher diffraction efficiency than the Au-coated counterpart, yielding a range of approximately 40% to 70% (Palmer & Loewen, 2005 ▸). As shown in Fig. 7 ▸(*b*), the total transmission efficiency of the system in the high-resolution mode is theoretically in the range of 4% to 15%. Notably, the transmission is sensitive to the polarization state of the incident light, with horizontal polarization yielding higher throughput. Based on the product of the undulator radiation flux and the overall beamline transmission efficiency, the normalized photon flux within a 0.1% bandwidth is shown in Fig. 7 ▸(*c*). Across the entire photon energy range, the beamline delivers a flux exceeding 10^14^ photons s^−1^ (0.1% bandwidth)^−1^, which is approximately an order of magnitude higher than that of the UE112-PGM beamline at BESSY II. When the effect of the beamline’s energy resolution is further taken into account, the total photon flux—depicted in Fig. 7 ▸(*d*)—remains above 10^12^ photons s^−1^ throughout the energy range in high-resolution mode.

**Figure 8:**
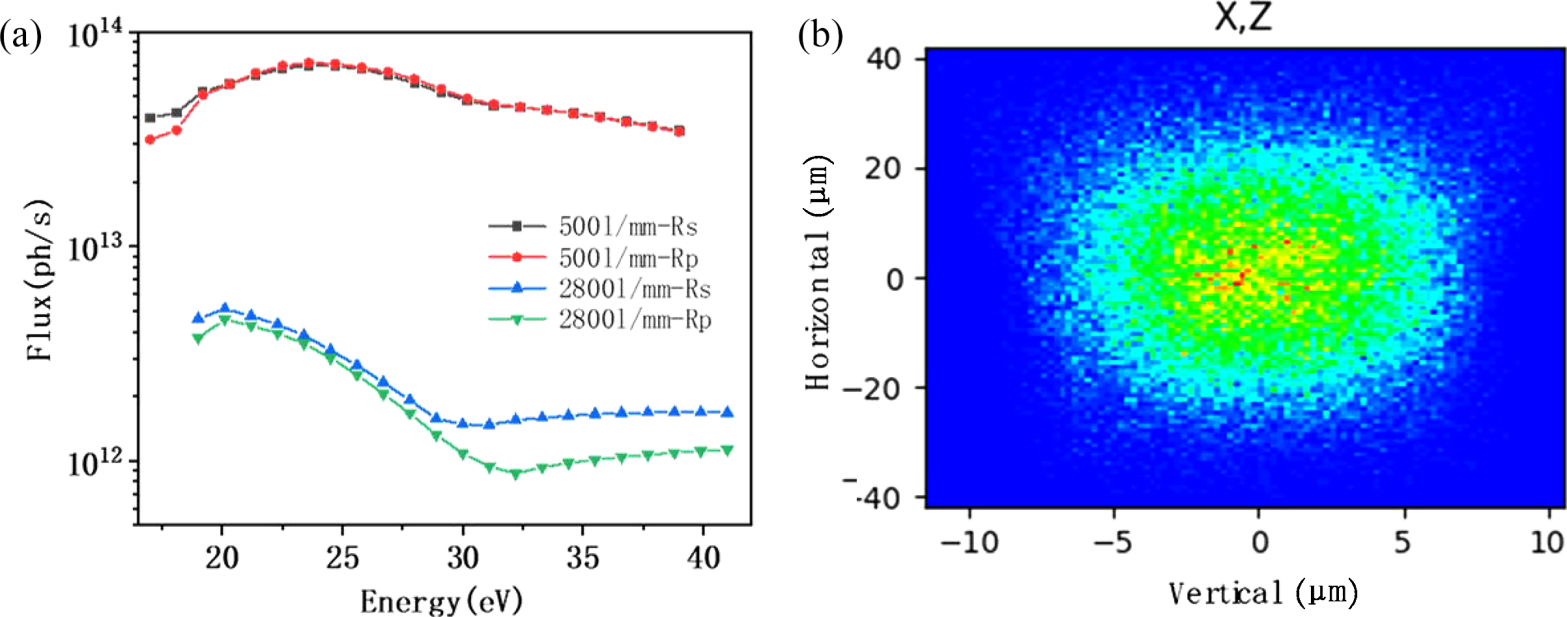
(*a*) Comparison of photon flux in high-resolution and high-flux modes. (*b*) Simulated spot size at sample point B when the vertical opening of the exit slit is 10 µm.

In the high-flux mode, the reflectivity of the plane mirror M2 remains essentially unchanged across the relevant photon energy range, as shown in Fig. 6 ▸(*d*). Consequently, the key factor influencing the enhancement of photon flux lies in the diffraction efficiency of the gratings. As illustrated in Fig. 7 ▸(*a*), a comparison between the diffraction efficiencies of VPG1 and VPG3 reveals that the latter exhibits approximately three times higher diffraction efficiency. However, this improvement comes at the expense of spectral resolution, which is reduced by a factor of about five. As a result, the overall photon flux in the high-flux mode increases by nearly an order of magnitude compared with the high-resolution mode, as depicted in Fig. 8 ▸(*a*). At a photon energy of 29 eV, the theoretical photon flux can reach up to 5 × 10^13^ photons s^−1^.

### 4.4. Expected focus size

For this beamline, vertical focusing at the sample position is achieved through the combined use of the toroidal M1, the VPG and the ellipsoidal mirror M4. Due to the use of the exit slit to define the energy resolution, the final vertical beam size at the sample is predominantly determined by the vertical aperture of the exit slit and the demagnification factor of M4. In high-resolution mode, a typical vertical slit opening of 10 µm is employed. The demagnification ratios of M4B and M4A are approximately 1:1 and 1.3:1, respectively. Since M4 is a horizontally reflecting mirror, its sagittal slope error has a negligible effect on the vertical beam size. Consequently, at endstation B, the vertical beam size at the sample position closely replicates the vertical opening of the exit slit, while, at endstation A, the vertical beam size is reduced by approximately 30% due to the higher demagnification. In the horizontal plane, focusing is accomplished sequentially by the toroidal mirror M1, the elliptical cylindrical mirror M3 and the ellipsoidal mirror M4. As all three mirrors reflect in the horizontal direction, their tangential slope errors have a significant influence on the horizontal beam size at the sample position. As shown in the optical simulation results in Fig. 8 ▸(*b*), the horizontal spot size at sample point B is approximately 30 µm, while at sample point A it is reduced to around 20 µm.

### 4.5. Suppression of higher-order harmonics and space-charge effects

Because undulators inevitably generate higher-order harmonic radiation, such components can introduce background signals and unexpected peaks in ARPES measurements. To suppress higher-order harmonics, we have reserved space downstream of the monochromator for the installation and adjustment of filter assemblies. Appropriate filters are selected for the required photon energy range to remove the unwanted harmonics. Fig. 6 ▸(*f*) shows the transmittance of several representative filter films across different photon energy ranges: Mg films are used for 17–40 eV, Al films for 24–40 eV, Sr films for 10–15 eV and In films for 11–17 eV. In addition, the grazing incidence angle of the monochromator’s plane mirror (M2) ranges from 16° to 26° (Fig. 3 ▸). Within this angular range, the reflectivity of a Si substrate decreases sharply above 40 eV, and the diffraction efficiency of the grating also drops significantly, thereby providing effective suppression of higher-order harmonics. These combined measures ensure that higher-order radiation will not significantly impact the performance of low-energy ARPES experiments. By selecting the appropriate filter at each photon energy, we estimated the photon flux ratio between the strongest higher-order harmonic (the third harmonic) and the fundamental harmonic using ray-tracing simulations. At a fundamental photon energy of 10 eV, the ratio is approximately 10^7^:1; at 12 eV, the ratio exceeds 500:1; and at 20 eV, the ratio is about 10^5^:1.

With the continuous enhancement of source brightness and the inherent temporal structure of the radiation, a fundamental limitation emerges when pursuing ultimate energy resolution. Whether employing laser systems that generate ultraviolet pulses or synchrotron radiation sources delivering brilliant undulator radiation, intense photon pulses inevitably create a relatively dense cloud of photoelectrons in front of the sample surface. During their transit to the energy analyzer, mutual Coulomb repulsion between these electrons distorts their initial energy and angular distributions, ultimately resulting in space-charge-induced energy broadening (Zhou *et al.*, 2005 ▸). The magnitude of this broadening is highly complex, being influenced by multiple parameters including photon flux, repetition rate, pulse duration, photon energy, spot size, sample properties and detection geometry (Hellmann *et al.*, 2009 ▸). This complexity necessitates subsequent experimental validation. Our storage ring operates at a repetition rate of 500 MHz with a pulse duration of 95 ps, representing a substantial advantage over quasi-continuous-wave laser sources (Table 1 ▸). The pulse duration is also longer than that of most storage rings, which are typically limited to ∼50 ps. Moreover, the top-up injection mode ensures exceptional beam current stability, offering a distinct benefit compared with non-top-up operation. As described below, we employ a wide-angle energy analyzer capable of collecting electrons over a 60° angular range, in contrast to conventional analyzers limited to 30°. In addition, the photon flux at our beamline (∼10^12^ photons s^−1^) is considerably lower than that of laser-based ARPES systems (>10^14^ photons s^−1^).

From a beamline design perspective, higher photon flux enhances the signal-to-noise ratio and improves measurement efficiency; however, if such high flux is not required, it can be readily reduced through beamline attenuation methods. The 30 µm spot size has been chosen to match the typical domain size of single-crystal samples, facilitating measurements across diverse sample types. If a larger spot size is desired, it can be achieved by adjusting mirror geometry or sample positioning. Finally, the energy broadening induced by the space-charge effect is effectively uniform within narrow energy ranges near the Fermi level, enabling post-acquisition correction through advanced data analysis approaches such as machine learning.

## 5. ARPES endstation

### 5.1. Compact ultra-low-temperature high-resolution ARPES

In ARPES systems, ultra-low temperatures are typically achieved using one of two approaches. The first employs a He-3 cryostat based on a Dewar structure, which can reach temperatures as low as ∼0.85 K (Borisenko, 2012 ▸). However, this method is costly and requires complex maintenance. The second approach utilizes a Dewar-free He-4 flow-type cryostat, which leverages the Joule–Thomson (JT) effect to achieve temperatures below 3 K. While this design is more compact and easier to maintain, it typically struggles to reach temperatures lower than 2.5 K (Shimojima *et al.*, 2015 ▸). At endstation B, we have developed a Dewar-free sample manipulator capable of reaching a base temperature of 1.5 K. This system eliminates the mechanical and operational complexity associated with conventional Dewar-based configurations and employs a two-stage JT cooling process to achieve temperatures below 1.5 K at the sample position. This capability enables high-resolution ARPES measurements of superconducting gaps in low-*T*_c_ superconductors.

### 5.2. High-efficiency large-angle spin-resolved ARPES

Endstation A is dedicated to measurements of the spin-resolved electronic structure of quantum materials. The system is equipped with a modern MCP/CCD-based energy analyzer that offers low electronic noise and a high signal-to-noise ratio. Unlike conventional ARPES analyzers, which typically support parallel detection with a limited angular range of ∼30°, this setup incorporates an extreme wide-angle lens capable of achieving parallel detection over a full 60° angular range. This significantly expands the accessible momentum space and enhances experimental efficiency. To enable spin-resolved measurements, a pair of twin very-low-energy electron diffraction (VLEED) spin detectors are integrated with the hemispherical analyzer in an orthogonal geometry (Tillmann *et al.*, 1989 ▸; Okuda *et al.*, 2008 ▸; Winkelmann *et al.*, 2008 ▸). This dual-detector configuration allows for full three-dimensional vector analysis of the electron spin polarization, enabling comprehensive characterization of spin textures in complex materials.

### 5.3. Vacuum-integrated sample preparation and processing system

**Figure 9:**
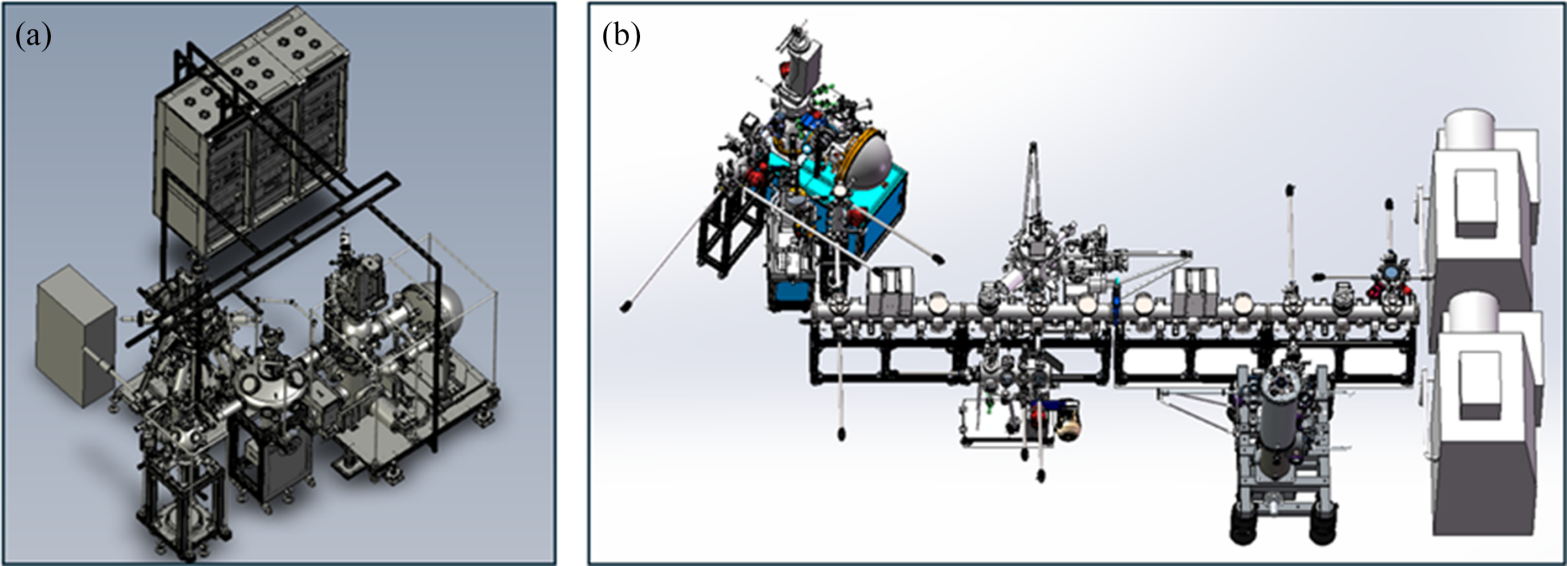
Engineering drawing of endstation B (*a*) and endstation A (*b*).

The ARPES station at UTEF is equipped not only with two advanced and functionally complementary electron structure measurement equipment but also with a comprehensive suite instrument for sample preparation, characterization and transfer. This infrastructure enables a fully integrated workflow—from synthesis and processing to *in situ* electronic structure analysis. Endstation B is connected via a radial distribution chamber (RDC) to an integrated oxide molecular beam epitaxy (OMBE) system and a vacuum transfer chamber that allows seamless sample exchange between endstations A and B. The OMBE system is equipped with a high-purity ozone generator, a reflection high-energy electron diffraction (RHEED) setup, a quartz crystal microbalance (QCM), multiple evaporation sources and a high-temperature, oxidation-resistant sample stage, making it well suited for the growth of high-quality oxide thin films and heterostructures. Endstation A is interfaced with a linear transfer line via a dedicated transfer node. Along this transfer line are several key modules, including a 2D material exfoliation chamber, a separate MBE system, a low-temperature scanning tunneling microscope (STM) and an inert-gas glovebox. In addition, the system includes an offline vacuum suitcase designed for sample exchange with external laboratories. This suitcase is capable of maintaining ultra-high vacuum and supporting magnetic fields above 8 T and temperatures as low as 4 K. The system layout is illustrated in Fig. 9 ▸.

In summary, the ARPES platform at UTEF constitutes a comprehensive, state-of-the-art facility for electronic structure research, offering end-to-end capabilities across sample growth, processing and measurement.

## 6. Summary

The Ultrafast Transient Experimental Facility at Chongqing University is developing an advanced ARPES beamline capable of 0.4 meV energy resolution within the 10–40 eV photon energy range, featuring photon flux over 10^12^ photons s^−1^, tunable polarization and ultra-low temperatures below 1.5 K. Leveraging a 0.5 GeV, 500–1000 mA storage ring, the design employs a dual-endstation setup for ultra-high-resolution and high-flux spin-resolved experiments. Optical optimization minimizes aberrations and achieves beam sizes around 30 µm (horizontal) and 10 µm (vertical), with overall transmission efficiencies of 4–15%. The beamline is scheduled for completion in 2026 and is expected to serve as a high-precision experimental platform for exploring quantum phenomena governed by the coupling of multiple degrees of freedom, addressing the pressing need for coordinated control over energy, spin, momentum and temperature in the multidimensional parameter space of high-resolution ARPES research—both at present and in the foreseeable future.

## Figures and Tables

**Table 1:** Main parameters of the UTEF storage ring and high-energy-resolution ARPES beamline

Storage ring energy, *E*_0_ (GeV)	0.5
Beam current, *I*_0_ (mA)	500–1000
Circumference, *C* (m)	76.78
Energy spread	3.7 × 10^−4^
Electron beam emittance, ɛ (nm rad)	17.27 (horizontal) × 1.727 (vertical)
Fundamental RF frequency, *f*_RF_ (MHz)	499.784
RMS bunch length with harmonic cavity, σ_*z*_ (mm)	12.2
Harmonic number, *h*	128
Photon energy range (high-resolution mode) (eV)	10–40
Photon energy range (high-flux mode) (eV)	20-40
Focused beam size (FWHM) (µm)	30 × 20
Energy resolution (*E*/Δ*E*)	>1 × 10^6^ @ 25 eV
Photon flux (photons s^−1^)	>1 × 10^12^ @ 25 eV

**Table 2:** Main parameters of the undulator

Type	EPU/APPLE-II
Period length (mm)	62
Number of periods	78
Magnet material	NdFeB with Br of 1.3 T
Standard magnet size (mm)	28 × 28 × 15.5
Vertical peak field, *B*_*y*0_ (T)	0.44
Horizontal peak field, *B*_*x*0_ (T)	0.69
Circular peak value (T)	0.37
Horizontal shift value (mm)	±31

**Table 3:** Optical parameters of the beamline

	M1	M2	VPG1	VPG2	VPG3	M3A/B	M4A	M4B
Energy covered (eV)	10–40	10–40	19–40	9.5–20	17–40	10–40	10–40	10–40
Blank material			Single crystalline silicon					
Dimension blank (l×w×h) (mm)	100×50×30	150×30×30	90×50×30	90×50×30	90×50×30	300×30×30	300×30×30	300×30×30
Optical surface (l×w) (mm)	80×20	130×20	Two strips, each 80×18			280×20	280×20	280×20
Include angle (°)	174	128–146				172	170	170
Blank plane	Toroidal	Plane				Elliptical cylinder	Ellipsoidal	Ellipsoidal
Parameters (mm)	*R* = 58662.8, ρ = 118.95	–	*g*_0_ = 2800 l/mm	*g*_0_ = 1400 l/mm	*g*_0_ = 500 l/mm	*r*_1_ = 10212	*r*_1_ = 2000	*r*_1_ = 2000
			*g*_1_ = 1.1421 mm^2^	*g*_1_ = 0.5711 mm^2^	*g*_1_ = 0.8775 mm^2^	*r*_2_ = 1500	*r*_2_ = 1520	*r*_2_ = 2000
			*g*_2_ = 2.013×10^−4^ mm^3^	*g*_2_ = 1.0065×10^−4^ mm^3^	*g*_2_ = 0.5395×10^−4^ mm^3^	Semi major axis = 5856	Semi major axis = 1760	Semi major axis = 2000
			*g*_3_ = −4.709×10^−8^ mm^4^	*g*_3_ = −2.355×10^−8^ mm^4^	*g*_3_ = −4.124×10^−8^ mm^4^	Semi minor axis = 273.01	Semi minor axis = 151.96	Semi minor axis = 174.31
Optical coating	Bare Au 40 nm	Bare Si	1st strip bare Si, 2nd strip Au 40 nm		Bare Si	Bare Si	Bare Si	Bare Si
Roughness RMS (nm)	0.5	0.5	0.5	0.5	0.5	0.5	0.5	0.5
Slope error RMS (µrad) (tangential/sagittal)	2.5/5	0.2/5	0.2/5	0.2/5	0.2/5	0.5/5	2.5/5	2.5/5

## References

[bb1] Borisenko, S. V. (2012). *Synchrotron Radiat. News* **25**(5), 6–11.

[bb2] Bosch, R. A., Kleman, K. J. & Bisognano, J. J. (2001). *Phys. Rev. ST Accel. Beams*, **4**, 074401.

[bb3] Cash, W. (1982). *Appl. Opt.* **21**, 710. 10.1364/AO.21.000710 20372522

[bb4] Chen, C., Nuckolls, K. P., Ding, S. H., Miao, W. Q., Wong, D., Oh, M., Lee, R. L., He, S. M., Peng, C., Pei, D., Li, Y. W., Hao, C. Y., Yan, H. R., Xiao, H. B., Gao, H., Li, Q., Zhang, S. H., Liu, J. P., He, L., Watanabe, K., Taniguchi, T., Jozwiak, C., Bostwick, A., Rotenberg, E., Li, C., Han, X., Pan, D., Liu, Z. K., Dai, X., Liu, C. X., Bernevig, B. A., Wang, Y., Yazdani, A. & Chen, Y. (2024*a*). *Nature* **636**, 342–347. 10.1038/s41586-024-08227-w PMC11634764 39663492

[bb6] Chen, X. Z., Wang, L., Ishizuka, J., Zhang, R. J., Nogaki, K., Cheng, Y. W., Yang, F. Z., Chen, Z. H., Zhu, F. Y., Liu, Z. T., Mei, J. W., Yanase, Y., Lv, B. Q. & Huang, Y. (2024*b*). *Phys. Rev. X* **14**, 021048.

[bb7] Eyers, A., Heckenkamp, Ch., Schäfers, F., Schönhense, G. & Heinzmann, U. (1983). *Nucl. Instrum. Methods Phys. Res.* **208**, 303–305.

[bb8] He, Y., Vishik, I. M., Yi, M., Yang, S. L., Liu, Z. K., Lee, J. J., Chen, S. D., Rebec, S. N., Leuenberger, D., Zong, A., Jefferson, C. M., Moore, R. G., Kirchmann, P. S., Merriam, A. J. & Shen, Z. X. (2016). *Rev. Sci. Instrum.* **87**, 011301. 10.1063/1.4939759 26827301

[bb9] Heimann, P. A., Koike, M., Hsu, C. W., Blank, D., Yang, X. M., Suits, A. G., Lee, Y. T., Evans, M., Ng, C. Y., Flaim, C. & Padmore, H. A. (1997). *Rev. Sci. Instrum.* **68**, 1945–1951.

[bb10] Hellmann, S., Rossnagel, K., Marczynski-Bühlow, M. & Kipp, L. (2009). *Phys. Rev. B* **79**, 035402.

[bb11] Hoesch, M., Kim, T. K., Dudin, P., Wang, H., Scott, S., Harris, P., Patel, S., Matthews, M., Hawkins, D., Alcock, S. G., Richter, T., Mudd, J. J., Basham, M., Pratt, L., Leicester, P., Longhi, E. C., Tamai, A. & Baumberger, F. (2017). *Rev. Sci. Instrum.* **88**, 013106. 10.1063/1.4973562 28147670

[bb12] Horie, T., Takakuwa, Y. & Nobuo Miyamoto, N. M. (1994). *Jpn. J. Appl. Phys.* **33**, 4684.

[bb13] Itou, M., Harada, T. & Kita, T. (1989). *Appl. Opt.* **28**, 146. 10.1364/AO.28.000146 20548441

[bb14] Kimura, S., Ito, T., Sakai, M., Nakamura, E., Kondo, N., Horigome, T., Hayashi, K., Hosaka, M., Katoh, M., Goto, T., Ejima, T. & Soda, K. (2010). *Rev. Sci. Instrum.* **81**, 053104. 10.1063/1.3425778 20515121

[bb15] Koike, M., Heimann, P. A., Kung, A. H., Namioka, T., DiGennaro, R., Gee, B. & Yu, N. (1994). *Nucl. Instrum. Methods Phys. Res. A* **347**, 282–286.

[bb16] Lagarde, B., Sirotti, F., Taleb-Ibrahimi, A., Miron, C. & Polack, F. (2013). *J. Phys. Conf. Ser.* **425**, 152022.

[bb17] Lei, T., Li, J., Lu, S., Wang, L., Yu, Q., Li, F., Wang, J., Wang, H., Ibrahim, K. & Zhang, K. (2022). *Phys. Rev. B* **105**, 115404.

[bb18] Lei, T., Tan, Y., Zhang, Z., Chen, S. & Feng, J. (2023). *Nucl. Instrum. Methods Phys. Res. A* **1048**, 168006.

[bb19] Li, J., Lei, T., Wang, J., Wu, R., Qian, H. & Ibrahim, K. (2020). *Appl. Phys. Lett.* **116**, 101601.

[bb5] Liu, G., Wang, G., Zhu, Y., Zhang, H., Zhang, G., Wang, X., Zhou, Y., Zhang, W., Liu, H., Zhao, L., Meng, J., Dong, X., Chen, C., Xu, Z. & Zhou, X. J. (2008). *Rev. Sci. Instrum.* **79**, 023105. 10.1063/1.2835901 18315281

[bb20] Lv, B. Q., Xu, N., Weng, H. M., Ma, J. Z., Richard, P., Huang, X. C., Zhao, L. X., Chen, G. F., Matt, C. E., Bisti, F., Strocov, V. N., Mesot, J., Fang, Z., Dai, X., Qian, T., Shi, M. & Ding, H. (2015). *Nat. Phys.* **11**, 724–727.

[bb21] Nahon, L., Alcaraz, C., Marlats, J., Lagarde, B., Polack, F., Thissen, R., Lepère, D. & Ito, K. (2001). *Rev. Sci. Instrum.* **72**, 1320–1329.

[bb22] Okuda, T., Takeichi, Y., Maeda, Y., Harasawa, A., Matsuda, I., Kinoshita, T. & Kakizaki, A. (2008). *Rev. Sci. Instrum.* **79**, 123117. 10.1063/1.3058757 19123555

[bb23] Padmore, H. A. (1989). *Rev. Sci. Instrum.* **60**, 1608–1615.

[bb24] Palik, E. D. (1998). *Handbook of Optical Constants of Solids.* Academic Press.

[bb25] Palmer, C. & Loewen, E. G. (2005). *Diffraction Grating Handbook* 6th ed. New York: Newport Corporation.

[bb26] Petaccia, L., Vilmercati, P., Gorovikov, S., Barnaba, M., Bianco, A., Cocco, D., Masciovecchio, C. & Goldoni, A. (2009). *Nucl. Instrum. Methods Phys. Res. A* **606**, 780–784.

[bb27] Poletto, L. & Frassetto, F. (2009). *Appl. Opt.* **48**, 5363–5370. 10.1364/AO.48.005363 19798376

[bb28] Rebuffi, L. & Sánchez del Río, M. (2016). *J. Synchrotron Rad.* **23**, 1357–1367. 10.1107/S1600577516013837 PMC5298219 27787241

[bb29] Reininger, R. (2011). *Nucl. Instrum. Methods Phys. Res. A* **649**, 139–143.

[bb30] Sasaki, S. (1994). *Nucl. Instrum. Methods Phys. Res. A* **347**, 83–86.

[bb31] Schäfers, F., Peatman, W., Eyers, A., Heckenkamp, Ch., Schönhense, G. & Heinzmann, U. (1986). *Rev. Sci. Instrum.* **57**, 1032–1041.

[bb32] Shimojima, T., Okazaki, K. & Shin, S. (2015). *J. Phys. Soc. Jpn* **84**, 072001.

[bb33] Strocov, V. N., Schmitt, T., Flechsig, U., Patthey, L. & Chiuzbăian, G. S. (2011). *J. Synchrotron Rad.* **18**, 134–142. 10.1107/S0909049510054452 PMC3133478 21335898

[bb34] Strocov, V. N., Wang, X., Shi, M., Kobayashi, M., Krempasky, J., Hess, C., Schmitt, T. & Patthey, L. (2014). *J. Synchrotron Rad.* **21**, 32–44. 10.1107/S1600577513019085 24365914

[bb35] Tanaka, T. & Kitamura, H. (2001). *J. Synchrotron Rad.* **8**, 1221–1228. 10.1107/s090904950101425x 11679776

[bb36] Tillmann, D., Thiel, R. & Kisker, E. (1989). *Z. Phys. B Condens. Matter* **77**, 1–2.

[bb37] Varykhalov, A. (2018). *J. Large-Scale Res. Facil.* **4**, A128.

[bb38] Winkelmann, A., Hartung, D., Engelhard, H., Chiang, C. T. & Kirschner, J. (2008). *Rev. Sci. Instrum.* **79**, 083303. 10.1063/1.2949877 19044342

[bb39] Xue, L., Reininger, R., Wu, Y.-Q., Zou, Y., Xu, Z.-M., Shi, Y.-B., Dong, J., Ding, H., Sun, J.-L., Guo, F.-Z., Wang, Y. & Tai, R.-Z. (2014). *J. Synchrotron Rad.* **21**, 273–279. 10.1107/S1600577513029093 24365949

[bb40] Zhou, X. J., Wannberg, B., Yang, W. L., Brouet, V., Sun, Z., Douglas, J. F., Dessau, D., Hussain, Z. & Shen, Z. (2005). *J. Electron Spectrosc. Relat. Phenom.* **142**, 27–38.

